# Promising Approach in the Treatment of Glaucoma Using Nanotechnology and Nanomedicine-Based Systems

**DOI:** 10.3390/molecules24203805

**Published:** 2019-10-22

**Authors:** Fidiniaina Rina Juliana, Samuel Kesse, Kofi Oti Boakye-Yiadom, Hanitrarimalala Veroniaina, Huihui Wang, Meihao Sun

**Affiliations:** 1 College of Chemistry and Life Sciences, Zhejiang Normal University, Jinhua 321004, China; younitra@gmail.com (F.R.J.); sanket.acharya4@gmail.com (H.W.); 2 School of Pharmacy, Shanghai Jiao Tong University, 800 Dongchuan Road, Shanghai 200240, China; kessejnr@yahoo.com (S.K.); otiboakye1000@gmail.com (K.O.B.-Y.); 3 Department of Pharmaceutics, School of Pharmacy, China Pharmaceutical University, Nanjing 211198, China; 4 State Key Laboratory of Modern Chinese Medicine, China Pharmaceutical University, Nanjing 210009, China; scholarha@yahoo.com

**Keywords:** nanotechnology, nanomedicine, drug delivery, MEMs, glaucoma, intraocular pressure, imaging

## Abstract

Glaucoma is considered a leading cause of blindness with the human eye being one of the body’s most delicate organs. Ocular diseases encompass diverse diseases affecting the anterior and posterior ocular sections, respectively. The human eye’s peculiar and exclusive anatomy and physiology continue to pose a significant obstacle to researchers and pharmacologists in the provision of efficient drug delivery. Though several traditional invasive and noninvasive eye therapies exist, including implants, eye drops, and injections, there are still significant complications that arise which may either be their low bioavailability or the grave ocular adverse effects experienced thereafter. On the other hand, new nanoscience technology and nanotechnology serve as a novel approach in ocular disease treatment. In order to interact specifically with ocular tissues and overcome ocular challenges, numerous active molecules have been modified to react with nanocarriers. In the general population of glaucoma patients, disease growth and advancement cannot be contained by decreasing intraocular pressure (IOP), hence a spiking in future research for novel drug delivery systems and target therapeutics. This review focuses on nanotechnology and its therapeutic and diagnostic prospects in ophthalmology, specifically glaucoma. Nanotechnology and nanomedicine history, the human eye anatomy, research frontiers in nanomedicine and nanotechnology, its imaging modal quality, diagnostic and surgical approach, and its possible application in glaucoma will all be further explored below. Particular focus will be on the efficiency and safety of this new therapy and its advances.

## 1. Introduction

Nanotechnology is a scientific nanoscale engineering technology conducted between the scales of nanometers [1]. In 1959, Richard Feynman, who is perceived as the father of nanotechnology, proposed the use of nanoscale machines in molecular and atomic modifications [1]. This submission was not properly examined until 1981 when the first scientist observed the nanoscale on a scan tunneling microscope [2]. Thereon after, our understanding and expertise of molecular and atomic scale modification became vastly revamped. Between the years 2000–2019, there has been a worldwide investment of approximately US$150.5 billion in the continuous research and development of nanotechnology. There has also been an increase in annual publications on nanotechnology since 1999 all the way to 2019, and this was mainly focused on drug delivery [2,3,4]. Nanotechnology is defined in two ways by Wagner et al. [5]: as technology using already existing knowledge on the human body and providing molecular aid for treatment and diagnostics, and it is further described as using nanostructure materials with divergent therapeutic effects. Kim et al. [6] further elaborated the description of nanomedicine in 2014 as using the aid of engineered nano-devices and nanostructures that operate extensively side by side at a single cell level for thorough examination, defense, control, repair, structuring, and enhancement at a molecular level of human biological systems, thus championing its medical goal.

The size of the particles used is seen as the main characteristic of nanotechnology [7]. Although using the traditional physics approach may not elaborately define matter interactions on a macroscopic level, nanoscale interfaces remain, however, primarily defined using quantum mechanics [8], therefore, nanomaterials have distinct chemical and physical characteristics diverse from materials at a macroscopic scale. One result of smaller particles is the expanded surface-to-volume proportion. Considering the per-unit size of a given material, a nanomaterial will offer a larger number of locales for synthetic responses than a macro scale material [8]. There are various nanomaterials that could be of extraordinary interest to the medicinal network because of their one-of-a-kind physical and compound attributes. Many nanomaterials give a gamut of useful options that embrace electrical conductivity, magnetic properties, biocompatibility, and biodegradability [5,9,10]. 

**Figure 1:**
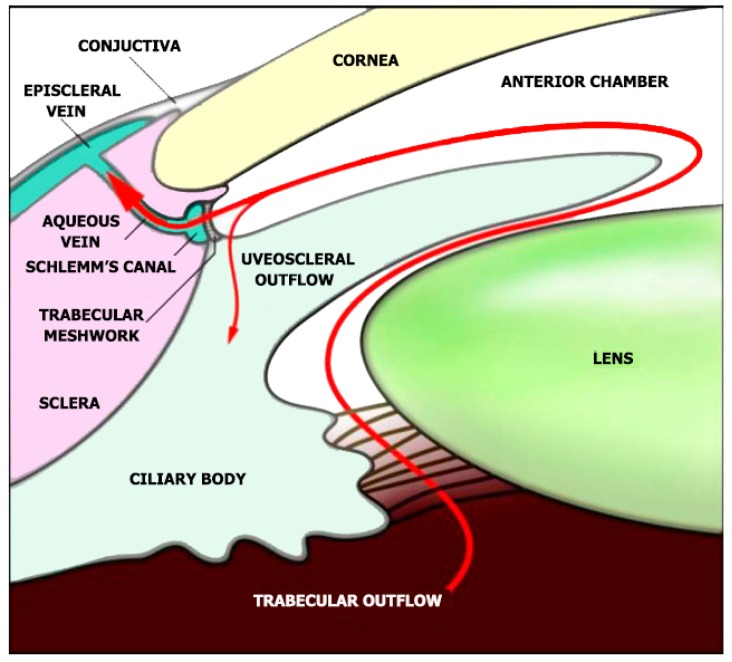
Aqueous humor indicated as the red line is produced by the ciliary body. The aqueous humor flows to the anterior chamber and leaves through the trabecular meshwork and Schlemm’s canal, then into the episcleral veins. The injection of nanoparticles is done through the anterior chamber and is perceived to follow the normal trabecular drainage pathway. Reproduced from ref [15,16] with permission from WILEY-VCH Verlag GmbH and Co. KGaA, Weinheim.

Glaucoma, a disease characterized by neurodegeneration which affects the retinal nerve fiber layers and the optic nerve head, is considered as one of the world’s leading causes of visual impairment. It is projected that by 2040, 111.8 million people worldwide will have the disease [11,12]. Amidst numerous subtypes, primary open-angle glaucoma (POAG) is the most typical type [13]. An important pathological risk for primary open-angle glaucoma is the degree of the intraocular pressure (IOP) [14]. The IOP is generated within the eye’s anterior chamber by resisting aqueous humor evacuation from the trabecular meshwork (TM) and the inner wall of the Schlemm’s canal (SC) (Figure 1) [15,16]. 

Ocular diseases have a direct effect on the sense of vision and value of human life. An assessment from thirty-nine countries found visual impairment affects 285 million people. Among these, 82% of the visually impaired are patients over 50 years [17]. A lot of improvements have been made in research on the management of ocular diseases and its pathogenesis [18,19,20,21]. However, because of the unique physiological obstacles and anatomic structures of the eye, the diagnostics and therapy of these diseases may lead to low potency and lack of specificity. Presently, therapy may only sometimes act to complete the restoration of eyesight or be able to detect serious eye diseases at an early stage [22]. Hence, there is a rapid increase in the attention shown on improving medical specificity and diagnostics and the medication for eye diseases. This review is centered on the advances in nanotechnology systems used in medical aid, therapy, and imaging in glaucoma therapy. Foremost, the precise anatomy and also the related limitations in ocular drug delivery systems are introduced. A number of the standard, as well as other drug delivery systems, are also explored. Next, in order to provide a more complex insight into the mechanism of nanosystems, many examples of nanotechnology and nanomedicine in the control of glaucoma are reviewed. Lastly, we present a summary of the nanotechnology angle and current obstacles in glaucoma diagnosis and therapy. Our review will not only provide incentive for superior design of glaucoma managements but also inspiration for further research. 

## 2. Anatomy of the Human Eye and its Constraints

**Figure 2:**
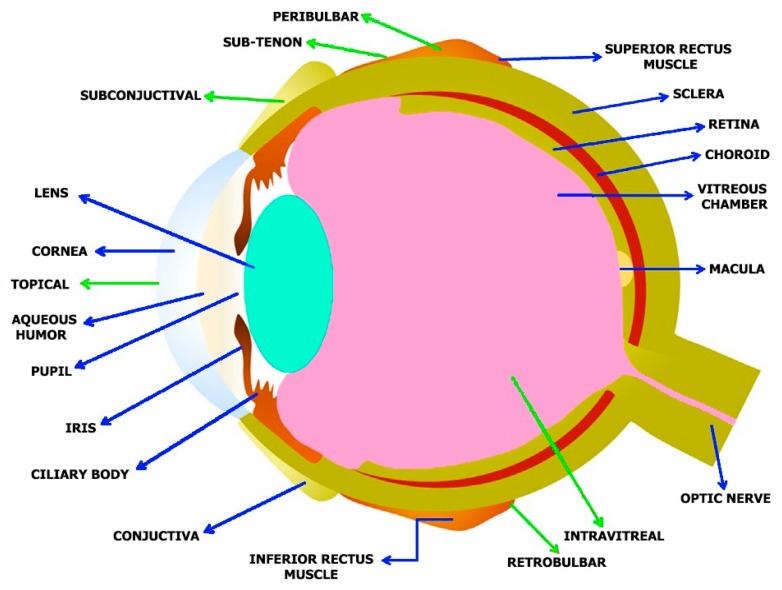
Anatomy of the human eye and administration routes for both nano based medicines and traditional drugs: Blue arrows indicates various eye structures and the green arrows indicate the various administration routes.

The human eye is an organ for vision and reacts to pressure and light [23]. It provides a three- dimensional mobile image usually colored in daylight. Cone cells and rod cells located in the retina help in sensing light and vision perception. Colors can be distinctively differentiated and depth perception is observable. The eye can distinctly tell apart about 10 million [24] colors and may be capable of detecting a single photon [25]. Akin to other mammalian eyes, the eyes of humans have non-image-forming photosensitive ganglion cells located in the retina, which allow light signal reception and regulate pupil size and repress the presence of the melatonin hormone and body-clock training [26]. The human eye has more of a coalesced two-piece unit shape as opposed to the conventional spherical shape proposition. There are two segments: the anterior, which encompasses the cornea, lens and iris, and; the posterior (Figure 2). 

The cornea, which is about 0.5 mm (500 μm) thick and 11.5 mm (0.3 in) in diameter, with a clear curvature connected to the larger posterior section comprised of the retina, vitreous humor, choroid, and the outermost white shell known as the sclera [23]. The posterior fragment makes up its remaining five sixths with a diameter of about 24 mm. The sclera and cornea are linked together by the limbus. The pigmented framework around the eye center and the darkly pigmented pupil is called the iris. The pupil size regulates the quantity of light penetrating the eyes, this is controlled by the iris dilator and muscles in the sphincter [23].

The human eye consists of three layers surrounding many anatomical structures [27,28]. The furthermost part is the fibrous tunic, containing the sclera and cornea. The middle part, which is called the uvea or vascular tunic, is composed of the iris, ciliary body, pigmented epithelium, and choroid. The inner part of the eye is the retina, which uses retinal vessels (interiorly) and choroid blood vessels for oxygenation [23].

The environment in the human eye has two major walls—the blood-aqueous and blood-retina walls. The former wall encloses the ciliary body’s pigment-less epithelium, which contains the Schlemm’s canal endothelium, iris epithelium, and the iris vessel endothelium. Cellular transports are controlled by the cell junction encompassing both the para and active types [29,30,31]. The latter barrier is fragmented into two, i.e., the outer layer and inner layer. The inner blood-retinal barrier carries the retinal vascular endothelium with tight junctions, while the outer blood-retinal barriers have a single layer of retinal pigment epithelium (RPE) with tight junctions [30,32]. The mentioned fragments constrict molecular penetrations into the intraocular chamber, leading to a deficiency in intraocular tissue treatment. Additionally, site-based drug delivery on the anterior ocular section is usually restricted by a corneal surface clearance mechanism and other pre-corneal elements, which could be the blinking of the eye, solution discharge, tear turnover, and film eye tearing [33]. The tear film of the human eye has a quick restoration period of about 2–3 minutes. Therefore, many site-based delivery drugs will be cleaned out in a short time after installation, e.g., precisely 2-3 minutes. If the drug solution which is topically applied is greater than 30 μL (the volume of the upper limit quartered in the cul-de-sac), a vast majority of drugs used may either be emptied by gravity-induced discharge or nasolacrimal drainage. Due to these obstacles and factors, the total drug efficiency of the medicine given is considerably less than 5%, thus rendering the bioavailability low [34,35].

## 3. Advancements of Nanomedicine in Glaucoma

**Figure 3:**
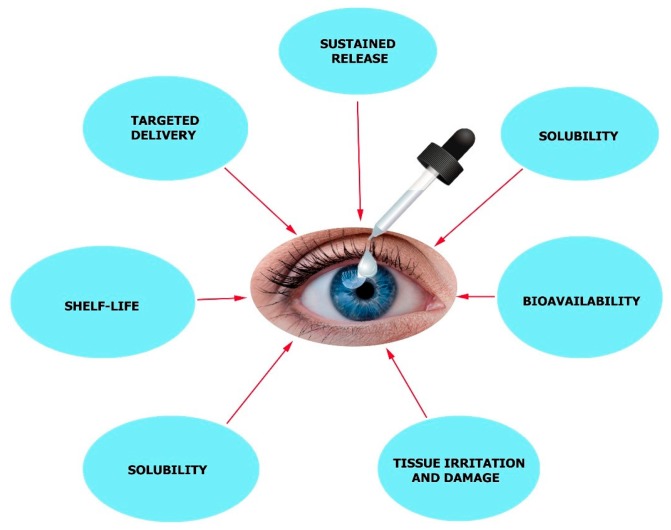
Major challenges in ocular disease treatment with eye drop formulations.

IOP-lowering agents are the most common form of anti-glaucoma treatment which is usually administered as eye drops [36]. Production of aqueous humor is suppressed by beta-blockers [37], carbonic anhydrase inhibitors [38], and alpha agonists which will reduce ocular pressure [39]. In order to enhance outflow, prostaglandin-like substances and other compounds can be used [40]. Patient compliance is very low as the medicine must be administered several times. There are other factors to be considered, like cornea permeability, tear turnover, the bioavailability of the medication, and short drug action duration. In order to overcome these limitations associated with this treatment, a more effective drug delivery system must be employed. It is in this case that nanotechnology-based delivery systems can be useful for newly developed drugs, which will provide sustained release, better bioavailability, an accurate dosing regimen, negligible irritation to tissues, lengthier shelf-life, targeted delivery, and enhanced solubility properties (Figure 3) [41,42].

**Figure 4:**
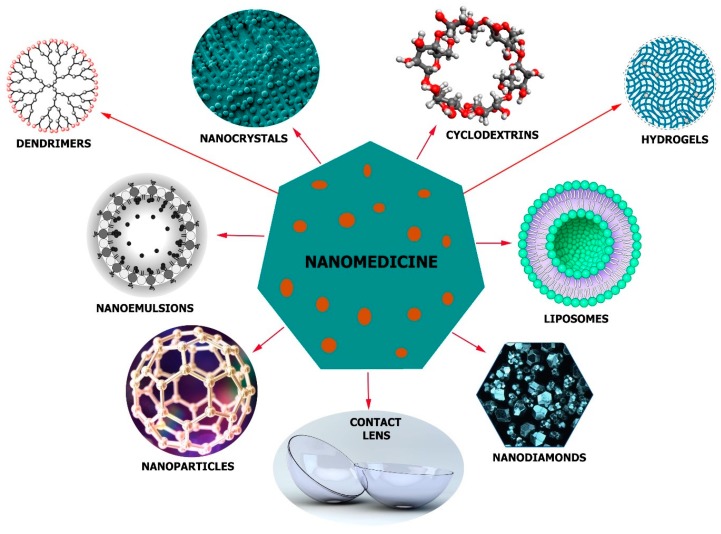
A scheme showing the structures of some nano-formulations used in glaucoma treatment: Nanoparticles with the ability to pass through biological barriers, nanoemulsions with increased drug solubility and membrane penetration, nanodiamonds with accessible surface area and tailorable surface chemistry, hydrogels, and nanocrystals for the conveyance of poorly water-soluble medicines, dendrimers, and liposomes capable of carrying hydrophobic and hydrophilic medicines, contact lens capable enzyme triggered release and cyclodextrins with the ability to form inclusion complexes enclose the lipophilic drug without changing its molecular structure.

The nano delivery systems can make use of nanoparticles (NPs) [43], nanoemulsions [44], nanodiamonds (NDs) [45], nanocrystals [46], liposomes [47], dendrimers [48], cyclodextrins [49], and other devices [50] together with contact lenses (Figure 4). The physicochemical properties (hydrophobicity, length, and stability), target tissue and the administration route determine which device and materials to use. Nonetheless, in order to get the desired effect, nontoxity of the delivery system, its biocompatibility, balance, and several other factors must be considered to suit the therapy.

Pilocarpine (a muscarinic receptor agonist), brimonidine (α_2_-adrenergic agonist), timolol and carteolol (beta-blockers), latanoprost (prostaglandin analog), and brinzolamide (carbonic anhydrase inhibitor) are modern anti-glaucoma medicines that have been employed in formulations as nanomedicines in glaucoma therapy [41,51,52]. In vivo rabbit studies used nano-drug preparations of timolol in the form of nanofiber patches and contact lenses and a significant reduction in the intraocular pressure was observed as compared with using drugs solutions [53]. In other studies, sustained drug release was observed when subconjunctival administration of anti-glaucoma microspheres was administered [54,55]. This was observed for three continuous months in in vitro experiments [56]. In addition, under in vitro conditions, 5% 1:1 timolol: propoxylated glyceryl triacrylate nanoparticles loaded onto a contact lens exhibited sustained release properties for up to one month [57]. 

### Triggered, Controlled and Sustained Drug Release in Glaucoma

During initial animal research in beagles, an effective reduction in the intraocular pressure was observed. A different and new contact lens layout containing vitamin E and timolol, known as ACUVUE TruEye, elicited a drastic reduction in the intraocular pressure [41,58]. Not very long ago, a device for the delivery of timolol was engineered and its lens was made to be enzyme-triggered [45]. Nanodiamonds were coupled to polyethyleneimine then chitosan was incorporated. A poly-HEMA matrix was fused to the nanodiamonds and fixed onto the lens. No drug release was seen when lysozyme was absent. It can be concluded, that the nanodiamonds ensured a controlled release of timolol when lenses were embedded with enzyme-cleavable polymers [45]. Alternatively, a nanofiber patch gadget containing polymers (polycaprolactone and polyvinyl alcohol) were also examined for the delivery of timolol after insertion of the cul-de-sac [59]. A 24-hour sustained drug release and intraocular pressure management for a period of 72 hours were observed in NZ (albino) rabbits using this new approach. 

**Figure 5:**
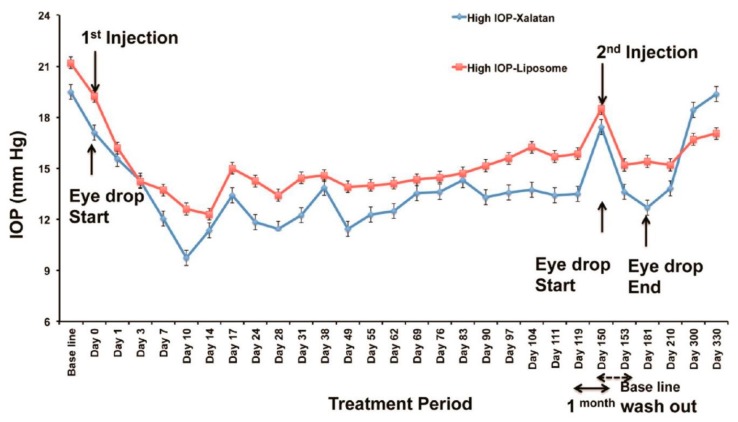
Comparative analysis between Xalatan eye drops and Latanoprost-loaded liposomes administered via the subconjunctival route in nonhuman primates. A great reduction of intraocular pressure was observed when a single injection of Latanoprost-loaded liposomes was done via the subconjunctival route for a period of 120 days in comparison with Xalatan eye drops. After a second injection, the intraocular pressure reduced further over another period of 180 days. Reproduced from [47] with permission from the American Chemical Society.

Natarajan et al. reported on a long-acting nanocarrier-based design for use in glaucoma therapy [47,60]. They reported a significant and successful improvement of a drug-encapsulated nanocarrier for decreasing intraocular pressure. A diseased nonhuman primate model was used and an explanation was given as to why in vivo sustained drug release was possible. Latanoprost, which is a prostaglandin analog, was used in this experiment and the carrier chosen was a uninamellar vesicle that was nanosized. Isothermal titration calorimetry explained the mechanism of action of this particular drug-nanocarrier interaction. Dynamic light scattering (DLS) and a Cryo-TEM were used to demonstrate that the shape of the liposomes was not altered and there was maximum drug loading of Latanoprost in the nanocarrier. As the first nanomedicine in glaucoma therapy, in vivo results clearly indicated good outcomes and confirmed the ability of this formulation to lower intraocular pressure (Figure 5) [47]. Considering the results, further work must be done to ensure nanocarriers are employed in glaucoma therapy using higher primates. A reduction in ocular pressure was observed in other experiments [61,62,63,64]. 

Recently, brinzolamide nanocrystal was synthesized using stable crystals [46,65]. A preliminary fast dissolution rate was observed indicating better bioavailability. Nonetheless, all the drug-loaded nanocrystals dissolved completely after one-hour. In comparing with the marketed product (49%), the intraocular pressure-lowering effect of the nanocrystals (79%) was very high in the animal models [46]. After a glaucoma surgical operation, a heat-sensitive injectable hydrogel for the administration of bevacizumab has been recently developed [66]. Polyethylene glycol–poly-ε-caprolactone–polyethylene glycol hydrogel after being injected into glaucomatous rabbit models exhibited no signs of increased intraocular pressure and infection. Comparing postoperative and preoperative values, the drug-loaded hydrogels showed a vast decrease in intraocular pressure within a period of three weeks.

**Figure 6:**
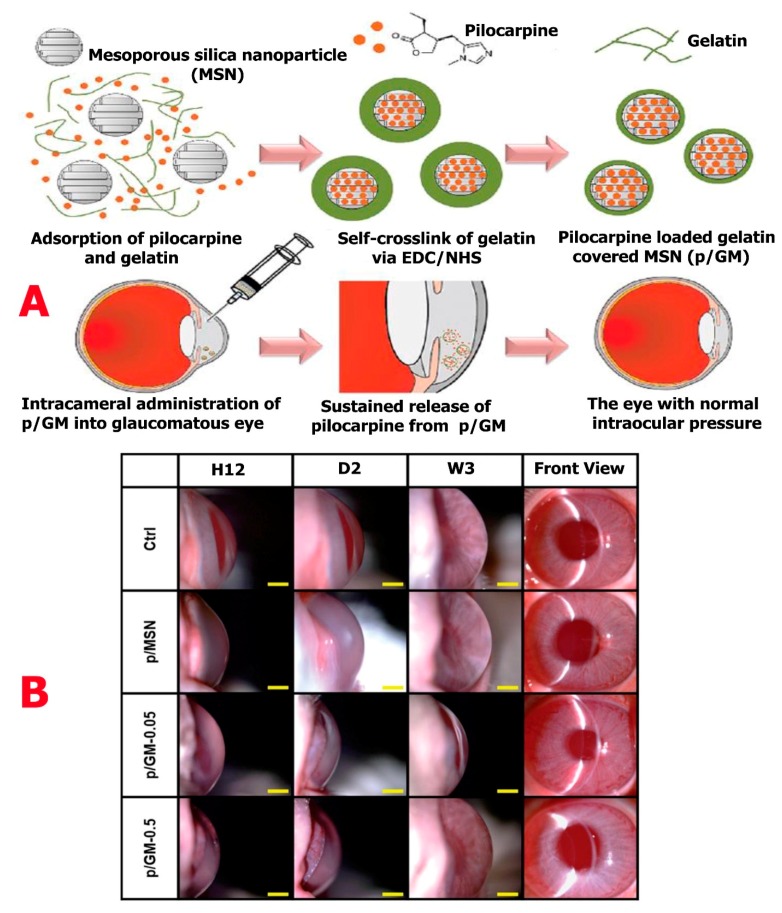
(**A**) Pilocarpine-loaded gelatin-covered mesoporous silica nanoparticles (p/GM) designed to reduce intraocular pressure. (**B**) Time-course slit-lamp biomicroscopy images of rabbit eyes injected with p/MSN, p/GM-0.05, and p/GM-0.5. After pilocarpine injection, the observation times are indicated as H12, D2, and W3 on a scale bar of 4 mm. Reproduced from [67] with permission from The Royal Society of Chemistry.

Liao et al. [67] in recent research, engineered pilocarpine-loaded gelatin-covered mesoporous silica nanoparticles (p/GM). These silica nanoparticles were administered via the intracameral route into the anterior chamber of the rabbit eye. This was done in order to reduce intraocular pressure (Figure 6A). Considering the in vitro release data, a long-lasting (36 days) and high percentage release (50%) of p/GM were observed. Intraocular pressure was maintained in the eye suffering from ocular hypertension for a period of 21 days (Figure 6B). For a sustained release behavior, the gelatin coat was controlled by varying the thickness on mesoporous silica nanoparticle, thereby enhancing the drug delivery of the nanoparticle. Looking at the in vivo and in vitro data, the synthesized p/GM-0.05 sample showed an extended-release profile and the potency to reduce intraocular pressure. 

Hassan et al. [68] in new research, studied the use of cationic nanoparticle (leciplex) in carvedilol delivery to ocular surfaces as a glaucoma therapy since other research [69,70] illustrated that carvedilol had an effect on intraocular pressure. As such, the leciplex formulae filled with carvedilol were made with cationic surfactant (CTAB/DDAB), soy phosphatidylcholine (SPC), and characterizations were done. After the formula used was evaluated and compared with carvedilol solution in vivo-studies, nanoparticles in leciplex were observed to be spherical in shape with an entrapment efficiency of 95% in all formulae. The leciplex preparation of DDAB and SPC in a molar proportion of 1:1 had the tiniest particle size (16.04 ± 1.2 nm), coefficient of the highest apparent permeability of the cornea at 0.1157 cm/h, and a good zeta potential (53.9 ± 0.91 mv). Carvedilol-leciplex complex was capable of decreasing intraocular pressure in rabbits with hypertension to a regular range 30 mins after administration over a period of 24 hours. Nonetheless, the solution of carvedilol decreased intraocular pressure 60 mins after administration for a period of 6 h. Previous examinations of carvedilol-leciplex administered to rabbit eyeballs with glaucomatous eyes improved their retinal atrophy. Proving that leciplex had a noticeable improvement in carvedilol bioavailability and transcorneal permeation. 

**Figure 7:**
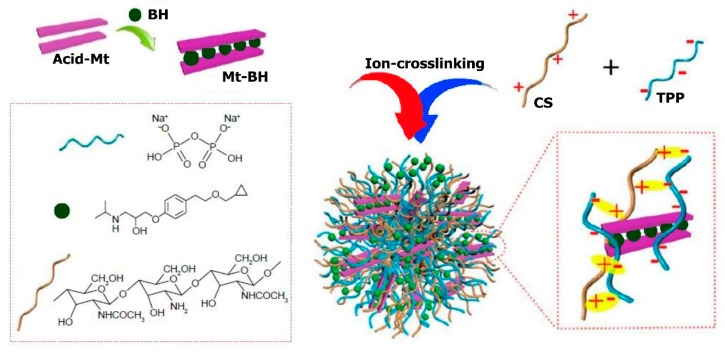
Schematic representation of the BH-Mt/CS nanoparticles preparation process. Reproduced from [71] with permission from Dovepress.

Li et al. [71] engineered novel nanoparticles to be used topically in eye-controlled drug delivery systems by insertion of betaxolol hydrochloride (BH) into the inner sheet of Na-montmorillonite (Na+Mt) and in addition, enchasing chitosan nanoparticles (Figure 7). The obtained nanoparticles had a diameter of about 460 ± 0.6 nm on average and a positive charge (+29 ± 0.18 mV). 

The in vitro research on the drug release profile showed regulated drug release patterns. The experimental studies of human immortalized cornea epithelial cells (iHCEC) and CAM-TBS found satisfactory ocular tissue tolerance. Additionally, nanoparticles were surprisingly found to have the ability to penetrate the iHCEC through cellular uptake observation by confocal layer scan microscopy (CLSM) measurements. Furthermore, iHCEC with many layers were used in in vivo preocular capacity of retention studies by corneal epithelial cells barrier stimulation showing that BH-Mt/CS nanoparticles compared with betaxolol hydrochloride solution had the ability to increase retention time. The pharmacokinetic studies of the eye through the technique of microdialysis sampling reported that MRT0−t of BH-Mt/CS nanoparticles and AUC0−t remained approximately 1.99-times and 1.75-times more than the betaxolol hydrochloride solution, reporting greater bioavailability. However, blood drug concentration studies by some researchers pinpointed that drugs with low levels could enter the blood, highlighting less system side-effects. Notably, the studies in pharmacodynamics disclosed that BH-Mt/CS nanoparticles could significantly drop glaucomatous rabbit’s intraocular pressure. Through this finding of chitosan/montmorillonite nanoparticles, it can be envisaged that BH-Mt/CS will be a potential carrier for betaxolol hydrochloride, thus making it an ideal candidate for glaucoma therapy. 

## 4. Nanosystems for Posterior and Anterior Glaucoma Therapy

Over the last decade, there has been development of a lot of traditional nanosystems for use in glaucoma anterior disease (Table 1). Contrary to anterior glaucoma diseases, diseases also affect the posterior section. Nanosystems currently used in the treatment of posterior glaucoma disease therapy are provided in Table 2. Many strategies based on nanotechnology in diagnosis of glaucoma diseases are further listed in Table 3.

## 5. Diagnostic and Therapeutic Glaucoma Nanodevices

### 5.1. Therapeutic Nanodevices

**Figure 8:**
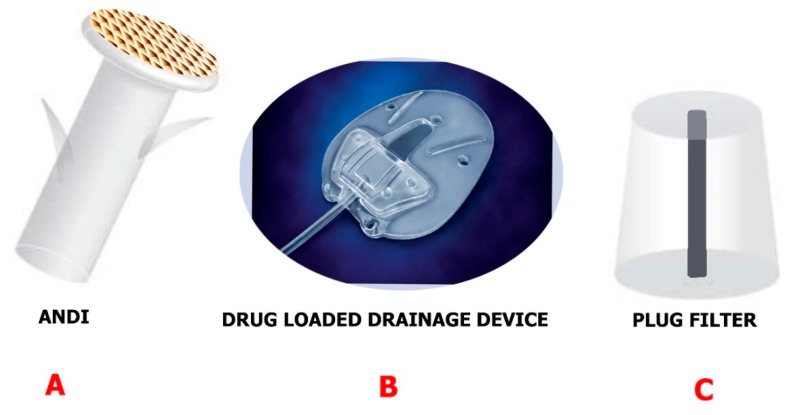
Examples of drainage devices engineered using nanotechnology approaches. (**A**) The ANDI is implanted in the sclera. Reproduced from [101] with permission from IEEE. (**B**) Drug-loaded drainage devices are manufactured with a film layer loaded with drug for continuous release. Reproduced from [99] with permission from Elsevier. (**C**) The plug filter is manufactured with a biodegradable polymer to eliminate postoperative hypotony. Reproduced from [103] with permission from IEEE.

Devices are mostly used in the drainage of glaucoma when trabeculectomy and IOP-lowering medication prove inefficient [50,94]. The mentioned devices give a different route for the aqueous humor to the collection plate beneath the conjunctiva from the anterior section of the eye. Those made by Ahmed, Molteno, Krupin and Baerveldt are the major devices commercially available for glaucoma draining [95,96,97]. Despite the fact that some differences exist between them, for example, their shape, size of plates, composition, and rigidity, they have successfully been able to contain and regulate IOP (within a period of five years) and there were no noticeable differences in vision preservation. Additionally, no noticeable differences were spotted in the total incidence of suprachoronical hemorrhage, postoperative hypotony, plate surrounding fibrosis, and inflammation [98]. Fibrosis is the main reason for the overall success decrease in five years from 40% to 50% in drainage implants. Even if antimetabolite injections like mitomycin C (MMC) and 5-fluorouracil (5-FU) have been found to both post and intraoperatively reduce complications [99], administration of injections to the bleb still causes a risk of infection and discomfort in patients. However, if the drainage device can be coated and used as a delivery vehicle, it could potentially have a greater effect on drug delivery systems (Figure 8B). Ponnusamy et al. [100] successfully manufactured a film of polylactic-co-glycolic acid (PLGA) with two layers which were loaded with 5-FU and MMC for drug release at a continuous rate. The studies conducted in vitro reported MMC was only stable on the surface, so 5-FU was loaded at the bottom. 5-Fluorouracil indicated to be released after three to five days continuously until day 28. After five days, the information obtained showed cell proliferation was inhibited by a COS-I cell culture model. This gives a promising use of glaucoma devices as an inflammation and fibrosis prevention method. 

Pan et al. [101] engineered an artificial nano-drainage system that can be implanted in the sclera of the eye and aid flow of aqueous humor. The nano-drainage implant known as ANDI (Figure 8A is composed of a polymeric shaft and a nanoporous membrane. There was clogging of proteins in the pores of the nanofiltration membrane, though the membrane was able to give the designed flow resistance. This promising nano-drainage system may be further improved by altering the surface chemistry.

A glaucoma valve that contains ferrofluid nanoparticles was developed by Paschalis et al. [102]. The ferrofluid was 10–100 nm in size and was magnetic in nature, had inert properties, and showed supramagnetic behavior. Until the magnetic pressure was lower than the liquid flow pressure, the ferrofluid served as a valve. Using x-ray diffraction, the stability of the device ascertained elicited no oxidation post air and water exposure. The intraocular pressure (11.8 ± 2 mm Hg) reduced after two weeks in comparison with contralateral control (14 ± 3 mm Hg) in in vivo rabbit studies. A plug filter was then engineered by Maleki et al. [103] to address postoperative hypotony (Figure 8C). Intraocular pressure is reduced when this filter is used but only for the short-term. Nonetheless, the hurdle of postoperative hypotony was overcome. To curtail this, the filter was engineered with polylactic acid (PLA) or PLGA. The plug was capable of reducing the intraocular pressure for a period of 15 days. 

Another work worth looking at was reported by Harake et al. [104]. They fabricated and engineered an antifouling micro glaucoma apparatus employing microelectromechanical methods. The device was composed of PEG-4000 and PEG-214. This polymer composition prevents swelling and clogging, thus making it an appropriate tool in preventing biofouling. The design was effective and resistant to *E.coli* and in vitro studies showed a lower specificity for protein absorption in comparison with polypropylene, glass, polymethyl-methacrylate, and polydimethylsiloxane. Further studies are being worked on to fully validate the efficiency of the device. 

### 5.2. Measurement of Intraocular Pressure

The extent of intraocular pressure plays a vital role in glaucoma therapy [105]. Leo et al. [106] developed a non-invasive, wireless, and soft silicone contact lens capable of detecting changes in intraocular pressure. A gauge sensor of platinum-titanium which was 170 nm thick and embedded in two layers was used. In order to power the sensor, a gold antenna, microprocessor, and an integrated circuit were fixed in the lens, this also aided in wireless communication via an external recording unit [106]. As the stress in the gauge increased, other mechanical forces compressed and altered the electrical forces present in the gauge. Leo et al. observed that the contact lens exhibited linear and reproducible intraocular pressure in the range (15–30 mm Hg) in enucleated pig eyes. Nonetheless, the contact lens was tested on 11 patients by Mossbockand and Faschinger and had limited success [106,107]. Aside from developing nanomaterials in drug delivery, nanotechnology has been able to be applied in the manufacturing of monitors capable of tracking intraocular pressure non-invasively [108,109,110,111,112]. 

A piezoresistive sensor capable of measuring intraocular pressure was engineered by Rizq et al. [113]. The sensor is capable of detecting changes in electrical resistance when stress is applied and had a radio frequency powering reverse telemetry as well as an interface circuit. In addition, Dresher et al. developed a compact circuit with low power which can be fixed in a wireless intraocular pressure monitoring system [114]. Applying the Bourdon tube technique, i.e., using a thin-walled, curved and hollow tube, other devices have been produced and are capable of measuring intraocular pressure [115,116,117]. Chen et al. used the Bourbon tube technique in developing a sensor that had a protective parylene membrane [115]. Extraordinarily, the device is capable of measuring intraocular pressure without depending on an external energy source. Though these sensors have an advantage, it should be noted that using the Bourbon tube is an invasive procedure and may uncomfortable for some patients [115,118]. All this research and work done serves as a good platform for wireless and implantable devices to be employed in patients with glaucoma and ocular hypertension in the future. 

### 5.3. Imaging

Disc photography initiated the role of imaging in glaucoma, thus enabling comparison and recording of qualitative changes in the optic nerve over a period of time [119]. Clinicians can have oversight in early changes in the optic nerve head (ONH) if they solely rely on photography, as that will be subjective analysis. For this reason, improvements have been made in fabricating stereoscopic images by means of flicker photography, which when employed, improves interobserver agreement on the neuroretinal rim width [120]. For diagnosis and management of glaucoma, the most widely used model is digital evaluation in the form of optical coherence tomography (OCT)[121,122,123,124,125]. Examination of the retinal nerve fiber layer (RNFL) using spectral-domain OCT (SD-OCT) is a good means of differentiating a glaucomatous eye from a healthy eye [126].

For a better understanding of biological pathways, nanoparticles have been employed in fluorescent imaging in glaucoma therapy [73,127,128]. Tam et al. used CdSe/ZnS core/shell nanoparticles which are quantum dots to trace lymphatic drainage in the eye of mice [129]. Unlike other conventional techniques, quantum dots are ideal for non-invasive in vivo imaging as a result of their tunable emission spectra, narrow emission sequence, and broad spectrum of excitation as well as a high photobleaching threshold [130]. The emission spectra may vary accordingly by altering the diameter of the quantum dots, thus allowing good adaption in several instances [129]. The quantum dots showed effective drainage via the skin and peritoneal spaces, and also enabled visualization of a freshly labeled lymphatic drainage path for fluid exiting the eye [129]. 

### 5.4. Surgical Implants

Surgical implants are capable of delivering drugs to the eye over a very long period of time. There are already implants for the long-term delivery of steroids. Examples include Retisert [131], an intravitreal implant that conveys fluocinolone acetonide for chronic uveitis over a period of 30 months [132], and Ozurdex (Allergan), a dexamethasone implant capable of conveying the drug over a period of six months via the intravitreal route of administration [133]. Surmodics I-vation implant, a triamcinolone acetonide coated implant with helical screws, is already available for delivering drugs intravitreal and has already undergone a Phase I clinical trial [132]. The use of surgical implants is not totally safe or cost-effective due to: the high cost; invasiveness of initial surgery; and should any adverse effect occur after the surgery, the complexity of removing the surgical implant. A vast number of glaucoma patients are mostly drawn back from undergoing surgery as a result of its associated risks [134]. 

Surgical implants may nonetheless provide a good platform for the delivery of neuroprotective medicines, as they are capable of delivering neuroprotective medicines to the retina effectively over a long period. For instance, the delivery of ciliary neurotrophic factor (CNTF)[135] can be done from a rice-sized implant through encapsulated cell technology for a period of one year [136]. The ciliary neurotrophic implant has undergone Phase I clinical trials for retinitis pigmentosa. All the patients used in the study showed high tolerance and enhanced visual acuity [137]. For a drug delivery system to be termed as ideal for glaucoma, it must be capable of ensuring a sustained release of the drug over a period of 3–4 months [138]. The 3–4 months drug release period will be ideal to ensure good results are obtained for follow-up evaluations. 

#### Refined Surgical Implants

Surgical implants are capable of delivering ocular medications over a long period of time. Several refined implants are coming up aside of the ones already available. If possible, one would prefer self-medication of ocular drugs to lower intraocular pressure and expect the drug to last for about 3–4 months just as that delivered by the surgical implants [139]. One innovative method is to implant a reservoir system in the subconjunctival space. Electrolysis that creates bubbles and pushes the medicine pot of the reservoir of the device is possible using a microelectromechanical system (MEMS) permitting multiple drug loading [140,141]. There are some similarities between the surgical steps and available glaucoma drainage devices, as the implant can be reloaded a lot of times and showed high tolerance in rabbit studies [142]. This is promising for the delivery of both small and large neuroprotective molecules, for example, growth factors [143,144].

An additional benefit of the microelectromechanical system is that by controlling electrolysis, the rate of drug release from the device can be regulated. Via an active delivery system, any clinician can alter the delivery rate upon assessment [145,146]. With very little modification, multiple drugs can also be administered via the intravitreal route using this device. In order to evaluate the sustained release function of the device and its stability, long term studies must be done. A major demerit and risk is that this implant must be surgically implanted in the eye, which poses some long term challenges [147]. 

## 6. Replacement and Regeneration of the Trabecular Meshwork (TM)

The primary drainage system for aqueous humor is the trabecular meshwork (TM), and it works as the major structure for controlling intraocular pressure [153]. This tissue has a sheet-like extracellular matrix (ECM) covered with endothelial-like and beams. Present are irregular inter-trabecular spaces making up the juxtacanalicular region, adjacent to the Schlemm’s canal (SC), and in between the sheets and beams. Prior to aqueous humor passing through the Schlemm’s canal (SC) and juxtacanalicular, the outer layer of the trabecular meshwork, which is phagocytic in nature, filters cell debris. Numerous fluctuations have been observed in the structure and function of the trabecular meshwork for a glaucomatous eye. This includes herniation in the extracellular matrix, variations in the levels of protein in the extracellular matrix, and improved action of calcification markers [154,155].

One of the recommended approaches for TM-focused treatments is gene therapy. Over the years, gene transduction has been made possible via the generation of recombinant adenoviral (Ad) vectors [156]. A dominant-negative RhoA gene carried by an adenoviral vector was effective in inactivating RhoA and lowering intraocular pressure in organ cultures. Actin cytoskeleton reorganization ceased as a result of inactive RhoA incapable of activating Rho kinase (ROCK). In this regard, intraocular pressure was reduced due to cellular relaxation in the trabecular meshwork [157], thus making ROCK inhibitors potentially beneficial in glaucoma therapy. It is possible to transduce genes using adenoviral vectors. To do this, an adenoviral vector carrying the matrix metalloproteinase 163 gene [158] controlled by glucocorticoid response elements was engineered, and it was observed that it could effectively lower intraocular pressure in a glaucomatous steroid sheep model [159]. 

Cell-based restoration of the trabecular meshwork tissue is a good alternate strategy because of its promising results in decreasing disease-related trabecular meshwork cells. To this effect, TM cells have been studied and were shown to localize in trabecular tissues after injection into the anterior capsule of mice for up to four months. This was done to ascertain its localization and differentiation into functional trabecular meshwork cells [160]. The presence of the expressed TM marker protein CHI3L1 and cell differentiation was detected in week one. This research served as a promising outcome for cell-based therapies and acts as a platform for TM tissue-based treatment.

Likewise, a biomimetic inner wall of the Schlemm’s canal that can serve as a tool for studying outflow physiology and drug screening was engineered with microfabrication techniques [161]. Furthermore, to test biomechanical roles and the effect of various therapeutic agents, artificial trabecular meshwork tissues are a good candidate. Micropatterned porous SU-8 scaffolds coated with poly-Llysine or gelatin to enhance trabecular meshwork attachment and cultured with human TM-cells for a 14-day period was the first attempt in this regard [162] and others followed [163,164]. A gelatin-coated 12-μm SU-8 microstructure was concluded to be the best design for meshwork formation and cell growth using perfusion studies and immunohistochemistry [162].

**Figure 9:**
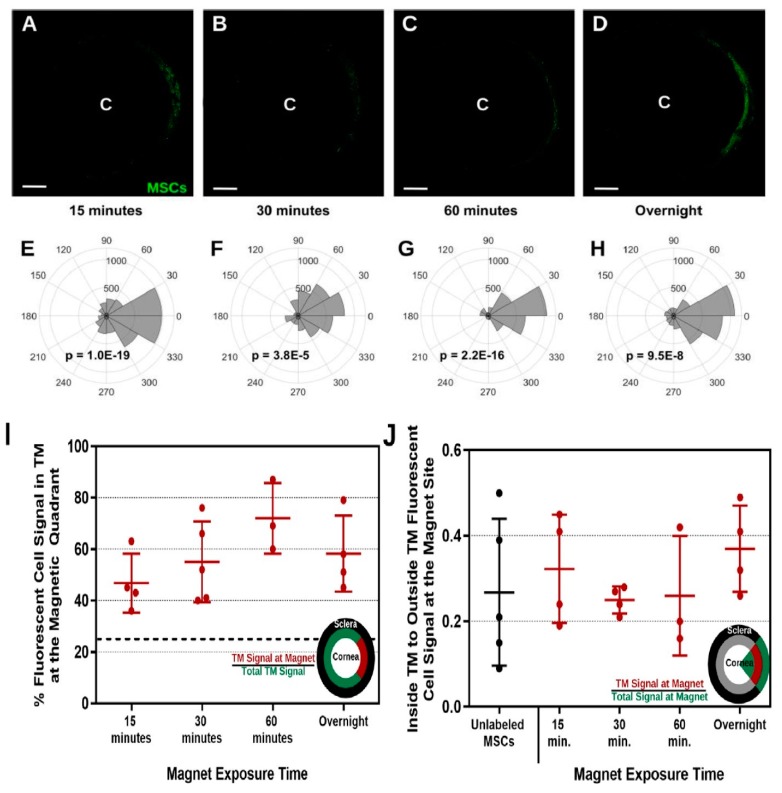
Outcome of Magnetic Field Exposure Time on directing 200 nm PBNC-MSCs to the trabecular meshwork. Figure (**A**–**D**) Shows anterior segment of the eye post mesenchymal stem cell delivery. There were bar magnets around the limbal region with *C* indicating the center of the human cornea. Figure (**E**–**H**) shows the intensity of total fluorescence within the trabecular meshwork area. P-values were calculated by means of Kuiper’s V test to evaluate if distribution was non-uniformly skewed in the direction of the focus of the magnet (0°). (**A**,**E**) 15 mins (*n* = 4 eyes), (**B**,**F**) 30 mins (*n* = 5 eyes), (**C**,**G**) 60 mins (n = 3 eyes), and (**D**,**H**) overnight (*n* = 4 eyes) with its exposure to the magnet are presented. Figure (**I**) indicates the percentage of fluorescent cell signal in magnetic quadrant in the trabecular meshwork, defined as the section encompassing 45° to −45°. The dotted line designates the expected value should there have been uniform cell delivery to the entire circumference of the TM (25%). Figure (**J**) Trabecular region fluorescence signal in comparison with non-trabecular fluorescent signal within the zone encompassing −45° to +45°. All evaluations between groups were not significant (*p* > 0.05) using one-way ANOVA with post hoc Tukey survey. Adapted from [165] with permission from Scientific Reports.

**Figure 10:**
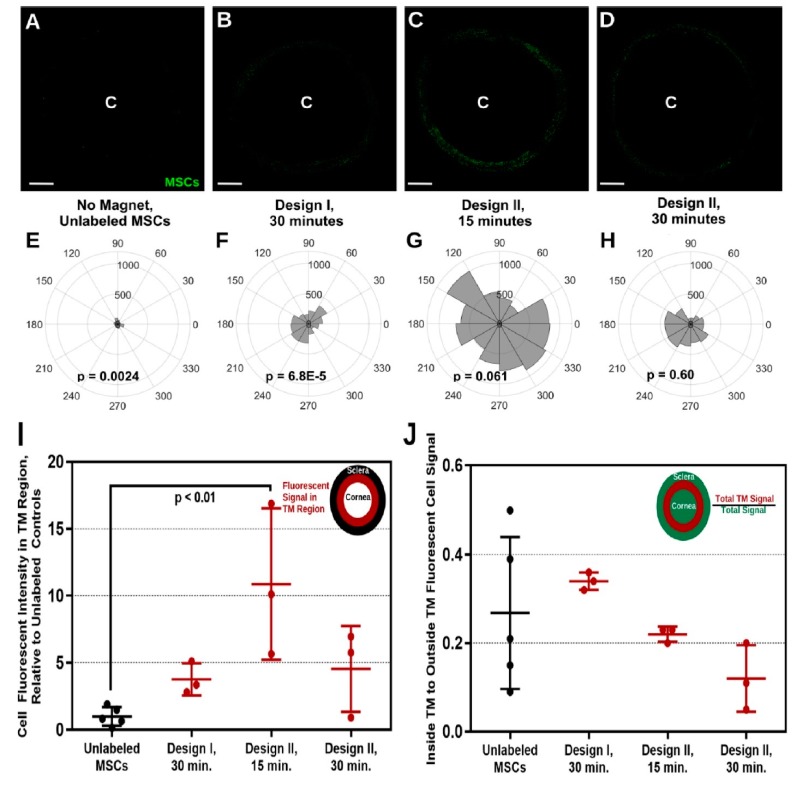
360° delivery of PBNC-MSCs using ring magnets. Figure (**A**–**D**) denotes injection of PBNC-MSCs into eyes by placing two types of ring magnets nearby the circumference for a diverse period of time with *C* indicating the center of the human cornea. Figure (**E–H**) shows the intensity of total fluorescence within the trabecular meshwork area. P-values were calculated by means of Rayleigh’s test to evaluate uniform delivery to the circumference (*p* < 0.05 denotes non-uniform delivery). Figure (**A**,**E**) no magnet (*n* = 5 eyes), (**B**,**F**) Magnet Design I for 30 mins (*n* = 3 eyes), (**C**,**G**) Magnet Design II for 15 mins (*n* = 3 eyes), and (**D**,**H**) Magnet Design II for 30 mins (*n* = 3 eyes). Figure (**I**) Cell signal quantification in the trabecular meshwork from injected mesenchymal stem cell eyes. Figure (**J**) Delivery of MSC specifically to the TM. Error bar indicates standard deviation with significant differences (*p* < 0.05) between groups calculated using post hoc Tukey analysis with ANOVA. Adapted from [165] with permission from Scientific Reports.

Snider et al. in 2018 [165] used magnetic steering to direct magnetic nanoparticles (Prussian blue nanocubes [PBNCs]) to the trabecular meshwork after injection of the nanoparticles into the eye’s anterior chamber. Comparing unlabeled cells and TM cells, PBNC-labeled stem cells displayed improved delivery after only a 15-minute exposure to a magnetic field (Figure 9). Further, the delivery of PBNC-labeled mesenchymal stem cells (MSCs) to the entire circumference of the trabecular meshwork was not possible without steering with a magnet. The multi-potency and viability of the mesenchymal cell were not affected by the PBNC (Figure 10). In conclusion, a targeted and high- efficiency delivery of stem cells to the trabecular meshwork was achieved using this approach, which are essential steps concerning regenerative medicine therapies in regulating ocular hypertension in glaucoma patients.

Dillinger et al. [16] proposed a novel, causative therapeutic idea which involves delivering small interfering RNA against CTGF via the intracameral route. A final layer of hyaluronan (HA) with layer-by-layer coated nanoparticles of 200–260 nm were synthesized. It was observed that the HA-coating allowed binding to TM and SC cells via CD44 and provided the nanoparticles enough mobility in ECM. The strength of the model was established by screening primary SC cells and TM ex vivo, in vivo, and in vitro. There was elevation of CD44 expression in healthy versus glaucomatous cells by about 2–6 times. CD44 is considerably involved in the cellular uptake of HA-coated nanoparticles. Compared to control nanoparticles, ex vivo organ culture of murine, human eyes and porcine validates up to threefold higher accumulation of HA and far enhanced permeation into the target tissue. In conclusion, a significant reduction of CTGF expression is brought about by gene silencing in primary human TM cells. Thus, RNA interference combined with HA-coated nanoparticles has great prospects in glaucoma therapy. 

## 7. Major Challenges, Expected Breakthrough, and Conclusions

### 7.1. Major Challenges 

Effective glaucoma drug treatment has numerous setbacks, all of which come under the headline of building a better eye drop [166,167]. Issues of how safe nano-based medicines are pose a major challenge in nanotechnology, though there have been great advancements in nano-based drug delivery [168]. The presence of particles, contaminants, and inflammatory variants in nanoparticle systems is a major setback [169]. As a result of Van der Waals forces, induced dipole force of attraction and hydrophobic interactions, numerous nanoscale materials show different characteristics. This allows interactions of proteins, biological membranes, and bodily fluids with nanoparticles possible. The shape and size of nanoparticles play a vital function in the effect on health. The size of the nanoparticles is inversely proportional to the surface area-to-mass ratio. This yields several reactive sites capable of undergoing bio interactions and uptake mechanisms [170,171]. 

The coating and surface charge of the nanoparticle determines its degree of effect on health, specifically with respect to toxicity. There is a better chance of positively charged particles undergoing maximum cellular uptake and interacting with intercellular membranes that are negatively charged. There is a higher probability of charged particles interacting with membranes and being cleared faster in comparison with neutral particles [170]. However, if released from the surface, the composition of the surface coating can cause adverse toxicity [172]. Particularly, the basis of biological interactions depends on the chemistry of the nanoparticles involved. Several nano-based systems have been used in the area of ophthalmology ranging from synthetic polymers to natural polysaccharides with their toxicity profiles very low [169]. Among these nano-based systems, PLGA, which is a biodegradable polymer, is considered the least toxic and approved by the US Food and Drug Administration (FDA). Likewise, at the cell penetration stage, polycaprolactone nano-capsules are also considered safe by the FDA [173]. Nano-micelles have also shown slight toxicity in biological systems [174] as well as acrylic copolymer Eudragit nanoparticles [169]. 

We focused on advances in nanotechnology and nanomedicine in glaucoma therapy in this review. Nanotechnology has demonstrated to be an effective tool in glaucoma therapy. In vitro and in vivo delivery of nanoparticles to treat glaucomatous eyes have been seen in several nanosystems with diverse cargos. Nonetheless, a lot of challenges exist and must be addressed in future research. Some include: (1) The rabbit’s eye possesses low tear production, high mucus production, high surface sensitivity and has a size comparable with the human eye [175]. These qualities are not precisely as the human eye, thus making mimicking difficult. (2) A number of the research is done for in vitro studies and has not focused much on in vivo studies. (3) Expansion and increase in the size of some nanoparticles after injection via the intravitreal route [176,177,178]. This can affect drug distribution and delivery efficiency. (4) Ocular disease-related biomarkers, molecular and cellular mechanisms, are not fully understood for targeted delivery [179]. All these must be addressed to fully curb and ensure more effective glaucoma therapy.

### 7.2. Expected Breakthrough 

Evidence is building that nanoparticles will serve as a promising drug delivery system, and it will not take much time until this technology reaches patients in the mainstream [180,181]. The quest for tailored medicine approaches in fighting glaucoma as a disease may be promising only through the development of nanotechnology platforms which encompasses molecular-level engineering. Nanoparticle engineering is a common thread for several imaging paradigms and drug delivery systems [182,183]. Nanotechnology has served, is still serving, and has promising prospects in glaucoma therapy and diagnosis. The eye is considered as one of the body’s most delicate organs and a perfect platform for drug and gene delivery because it bypasses systemic circulation. More than 1600 gene therapy clinical trials for ophthalmology are ongoing according to data from the Wiley Library [184]. Non-invasive routes of administration should be focused on using various nanomaterials and devices. Lastly, a system that combines therapeutic functions and diagnostics should be built to aid in visual tracking in glaucoma therapy. 

### 7.3. Conclusions

Several effective anti-glaucoma medicines and devices have been developed over the years. On the other hand, poor patient adherence, side effects, poor bioavailability, and inefficient delivery systems limit their clinical efficacy. Innovative and more competent delivery systems are being developed to improve patient adherence, reduce toxicity profiles, and side effects. Finally, these novel delivery systems for potential neuroprotective drugs and lowering intraocular pressure can initiate greater treatment possibilities and preservation of vision in glaucoma. Further investigations and research need to advance our understanding of the fundamentals of nanoparticles and encourage development of proper delivery routes for glaucoma therapy. Considerable clinical and basic research needs to be done to ensure progress in the direction of personalized medicine for refining treatment outcomes for patients with glaucoma [185].

## Figures and Tables

**Table 1:** The current and traditional strategies based on nanotechnology used in the therapy of anterior glaucoma disease.

Formulation	Role/Function	Cargo	Material Type	Size (nm)	Stage	Ref.
Nanoparticle	Exhibition of superior transfection potency at the anterior region of the eye.	Gene	Chitosan	~200	Preclinical stage	[72]
Nanowafer	High level of nontoxicity with a low dose and ability to efficiently treat corneal neovascularization as compared with eye drops.	Axitinib	Polymer	500	Preclinical stage	[73]
Nanosuspension	Increasing retention time and penetration in corneal tissues.	Diclofenac	Polymer	105	Preclinical stage	[74]
Hydrogel (Virgan)	Treatment of herpes simplex infection in the eye.	Ganciclovir	Polymer	Data not found	On the market	[75,76,77,78]
Nanoparticle	Increased anti-inflammatory effect via the topical route.	Flurbiprofen	Polymer	200–300	Preclinical stage	[79]
Nanoparticle	Improved the efficacy of the drug to aid in corneal graft rejection.	Dexamethasone sodium phosphate	Polymer	100–500	Preclinical stage	[80]
Nanoscale dispersed ointment	Improved efficacy of corneal surface restoration and tear film repairing.	Data not found	Polymer	100	Preclinical Stage	[81]
Nanoparticle	Inhibitory effect in rabbit models with a mild response using a low drug concentration. A higher volume of the drug penetrated the aqueous humor in comparison with eye drops.	Flurbiprofen	Polymer	100	Preclinical stage	[82]
Hydrogel	Improvement of drug bioavailability in the eye as well as retention time on the surface of the cornea.	Diclofenac	Polymer	Data not found	Preclinical stage	[83]
Liposome	High anti-cataract effect with enhanced superoxide dismutase activity and glutathione reduction.	Coenzyme-Q10	Polymer	100–200	Preclinical stage	[84]
Nanoparticle	Increase miotic response to 40%.	Pilocarpine	Polymer	83	Preclinical stage	[85]

**Table 2:** The current and traditional strategies based on nanotechnology used in the therapy of glaucoma posterior diseases.

Formulation	Role/Function	Cargo	Material Type	Size (nm)	Stage	Ref.
Nanoparticle	Provide sustained drug release via the subconjunctival route.	Latanoprost acid	Polymer	80	Preclinical stage	[47]
Hydrogel	Sustained drug release of Bevacizumab in SD rats for a period of four months.	Bevacizumab	Polymer	Data not found	Preclinical stage	[86]
Hydrogel (Timoptic-XE)	Treatment of glaucoma.	Timolol maleate	Polymer	Data not found	On the market	[87]
Liposome	Ability to pass through biological barriers after topical administration via annexin-A_5_ mediated endocytosis	Bevacizumab	Polymer	100–200	Preclinical stage	[73]
Hydrogel	Sustained drug release and decrease intraocular pressure as well as good compatibility with polymer.	Mitomycin C	Polymer	Data not found	Preclinical stage	[88]
Liposome	Entrapment of RPE cells and increased siRNA delivery by four-fold.	Gene	Polymer	130–230	Preclinical stage	[89,90]
Micelle	Sustain drug release for a period of one year after intravitreal injection in rat eyes.	Triamcinolone acetonide	Polymer	200–350	Preclinical stage	[72]
Dendrimer	Effective gene transfection in RPE cell lines.	Gene	Polymer	~50	Preclinical stage	[91]
Nanoparticle	Ability to prevent retinal degeneration and increase histological properties.	Gene	Peptide/ polymer	~180	Preclinical stage	[92]
Nanoparticle	Promotion of gene expression in RPE cells.	Gene	Polymer	~250	Preclinical stage	[93]

**Table 3:** Prospective nanotechnology-based approaches for glaucoma diagnostics.

Formulation	Role/Functions	Target	Material Type	Size (nm)	Stage	Ref.
Nanoparticle	Nanoparticles coated with calcium elicit slight damage and can be used in retina imaging.	Retina	Silver	80	Preclinical stage	[148]
Nanoparticle	Improvement of angiogenic vessels in a rabbit corneal neovasculature model.	Corneal neovascularization	Gold	~260	Preclinical stage	[149]
Nano-cage	Good optical resonance	Retina	Gold	35	Preclinical stage	[150]
Nanoparticle	Enhanced and clear fluorophores in eye imaging.	Intraocular cancer	Quantum dots	3–6	Preclinical stage	[129,151]
Nanoparticle	Diffusion of nanoparticles present in in vitro model of human vitreous humor.	Retinal detachment	Magnetic Nanoparticles (Fe3O4)	10	Preclinical stage	[152]
